# An Efficient CRT Based Algorithm for Frequency Determination from Undersampled Real Waveform

**DOI:** 10.3390/s23010452

**Published:** 2023-01-01

**Authors:** Yao-Wen Zhang, Xian-Feng Han, Guo-Qiang Xiao

**Affiliations:** College of Computer and Information Science, Southwest University, Chongqing 400715, China

**Keywords:** robust Chinese Remainder Theorem, frequency estimation, undersampling, error bound, sensor network

## Abstract

The Chinese Remainder Theorem (CRT) based frequency estimation has been widely studied during the past two decades. It enables one to estimate frequencies by sub-Nyquist sampling rates, which reduces the cost of hardware in a sensor network. Several studies have been done on the complex waveform; however, few works studied its applications in the real waveform case. Different from the complex waveform, existing CRT methods cannot be straightforwardly applied to handle a real waveform’s spectrum due to the spurious peaks. To tackle the ambiguity problem, in this paper, we propose the first polynomial-time closed-form Robust CRT (RCRT) for the single-tone real waveform, which can be considered as a special case of RCRT for arbitrary two numbers. The time complexity of the proposed algorithm is O(L), where *L* is the number of samplers. Furthermore, our algorithm also matches the optimal error-tolerance bound.

## 1. Introduction

Chinese Remainder Theorem (CRT) is a fundamental number theory result, which shows the reconstruction of a single integer *X* from its residues modulo multiple co-prime moduli. It has been extensively used in various applications, such as wireless sensor networks [1,2], coding theory [3,4,5,6,7], phase unwrapping [8,9], and frequency estimation from undersampled waveforms [10,11,12,13,14,15,16,17]. In particular, the CRT-based method enables one to estimate frequencies with exponentially smaller sub-Nyquist rates in a distributed setup. This could significantly reduce hardware cost [18,19]. In practice, errors may occur in the spectrum measurement, while CRT is known highly sensitive to residue perturbation [20]. Moreover, in some applications of multiple parameter estimation, we may need to recover multiple real numbers simultaneously. To this end, many works have been proposed during the last two decades to solve the two issues, which can be summarized as follows.

(i) Robustness: On the one hand, to make CRT robust against small errors in residues, Wang et al. introduced a common factor Γ as redundancy to the co-prime moduli $\{M_{1},M_{2},$$\cdots ,M_{L}\}$ in a form $\left\{m_{l}=M_{l}\Gamma |l=1,2,\cdots ,L\right\}$. This forms the foundation of the first closed-form Robust CRT (RCRT) for a single real number [20]. RCRT can recover the folding number ⌊X/Γ⌋ once the error in each residue is upper bounded by Γ/4. Hence, one can ensure the reconstruction error is upper bounded by Γ/4. The Γ/4 error tolerance bound is also proved to be tight in the follow-up works [21].
(ii) Residue Ambiguity: On the other hand, since the observed residues are unordered, there is no clear correspondence between *N* numbers $\left\{X_{i}|i=1,2,\cdots N\right\}$ and residues in each residue set $R_{l}=\left\{r_{i,l}|i=1,2,\cdots ,N\right\}$, l=1,2,⋯,L. Here, $r_{i,l}$ denotes the residue of $X_{i}$ modulo $m_{l}$. Thus, the residue ambiguity makes reconstruction much more complicated for multiple numbers. When N=2, Xiao et al. proposed a robust generalized CRT, addressing the residue ambiguity by carefully-designed quadratic symmetric polynomials [22]. It is shown that the correspondence between these two numbers and residues can be uniquely determined while the error bound is sacrificed to Γ/8 [23]. As shown in recent works [24,25]; theoretically, one can approach the optimal error bound Γ/4 independent of *N* when the least common multiple of moduli is sufficiently large. However, to the best of our knowledge, no existing polynomial-time algorithm matches the optimal bound.

In this paper, we focus on CRT based algorithm for frequency determination from the undersampled real waveform. The proposed method can be applied in a sensor network with low power and low transmission rates sensors [26,27] or Synthetic Aperture Radar (SAR) imaging of moving targets [28]. However, in the real waveform scenario, the CRT-based method encounters both the above-mentioned challenges, robustness, and residue ambiguity, simultaneously.

Notably, the real waveform sampling needs less hardware, i.e., only one Analog-to-Digital Converter (ADC) per sampling frequency is required in real waveform sampling rather than two ADCs in complex waveform [29]. However, existing CRT methods for the complex waveform cannot be applied to the real waveform directly due to the existence of the spurious peak [24]. In this paper, we set out to solve these mentioned issues. Our main contributions can be concluded as follows.

- We present the first polynomial-time closed-form RCRT for frequency determination from undersampled single-tone real waveform, which provides a feasible and efficient solution. Moreover, the proposed method fixes the gap in the CRT-based method for frequency determination for the real waveform case.
- By fully utilizing the prior knowledge of the real waveform, we reach the optimal error tolerance bound, i.e., Γ/4, which is twice better than the best-known robust generalized CRT proposed in [23].

The remaining content is organized as follows. In Section 2, we give an overview of the problem formulation. Section 3 details our closed-form reconstruction for the real waveform. In Section 4, we present some simulation results to support the theory. In Section 5, we discuss and interpret the simulation results. The conclusion is drawn in Section 6.

## 2. Problem Formulation

We first describe the frequency estimation model from the undersampled real waveforms.

### 2.1. Signal Model and Sampling

A sinusoidal waveform is defined as

(1) $$x\left(t\right)=A\cos\left(2\pi Xt\right)=\frac{1}{2}\left(Ae^{\left(2\pi jXt\right)}+Ae^{\left(-2\pi jXt\right)}\right),$$

where *A* denotes the amplitude, *X* represents the frequency. Sampling x(t) with *L* ADCs at frequency rates of $\left\{m_{l}|l=1,2,\cdots ,L\right\}$ [24,30], where $\max_{l}m_{l}<2X$, i.e., the sampling rates are below the Nyquist rate, we have

(2) $$x_{m_{l}}\left[u\right]=\frac{1}{2}\left(Ae^{\frac{2\pi jXu}{m_{l}}}+Ae^{\frac{-2\pi jXu}{m_{l}}}\right),\;u\in Z.$$

Applying the $m_{l}$-point Discrete Fourier Transform (DFT) to $x_{m_{l}}\left[u\right]$ [31,32], we obtain

(3) $$DFT_{x_{m_{l}}\left[u\right]}\left[k\right]=\frac{A}{2}\delta \left(k-{\langle X\rangle }_{m_{l}}\right)+\frac{A}{2}\delta \left(k-{\langle -X\rangle }_{m_{l}}\right).$$

Here, δ(*) is the the Kronecker delta function, i.e., $\delta (k-{\langle X\rangle }_{m_{l}})$ equals 1 when $k={\langle X\rangle }_{m_{l}}$ or 0 otherwise, where *k* represents a frequency bin and ${\langle X\rangle }_{m_{l}}$ denote the residue of *X* modulo $m_{l}$. Clearly, the locations of the spectrum peaks correspond to the residues ${\langle X\rangle }_{m_{l}}$ and ${\langle -X\rangle }_{m_{l}}$, which leads to two symmetric peaks over the frequency spectrum domain in the noiseless case. Thus, one can recover the frequency *X* with sampling rates (moduli) $m_{l}$ and the locations of the spectrum peaks (residues) ${\langle X\rangle }_{m_{l}}$ via CRT.

### 2.2. Noise Model and RCRT Procedure

In the following, we further consider the noisy case and review RCRT. Still, let $X_{i}\in \left\{X_{1},X_{2}\right\}$ represent the real number to be recovered, where $X_{1}>0$ and $X_{2}=-X_{1}$. The moduli are in a form $\left\{m_{l}=M_{l}\Gamma |l=1,2,\cdots L\right\}$, where $\left\{M_{l}\right\}$ are pairwise co-prime. ${\tilde{r}}_{i,l}={\langle X_{i}+\Delta_{i,l}\rangle }_{m_{l}}$ denotes the erroneous residue of $X_{i}$ modulo $m_{l}$, where $\Delta_{i,l}$ represents the underlying error such that $|\Delta_{i,l}|<\Gamma /4$. Moreover, $r_{i}^{c}={\langle X_{i}\rangle }_{\Gamma }$ denotes the common residue of $X_{i}$. ${\tilde{r}}_{i,l}^{c}={\langle X_{i}+\Delta_{i,l}\rangle }_{\Gamma }={\langle r_{i}^{c}+\Delta_{i,l}\rangle }_{\Gamma }$ denotes the erroneous common residue. In practical cases, ${\tilde{r}}_{i,l}^{c}$ is calculated from ${\tilde{r}}_{i,l}$ based on the number theory, i.e., ${\tilde{r}}_{i,l}^{c}={\langle X_{i}+\Delta_{i,l}\rangle }_{\Gamma }={\langle {\langle X_{i}+\Delta_{i,l}\rangle }_{M_{l}\Gamma }\rangle }_{\Gamma }={\langle {\tilde{r}}_{i,l}\rangle }_{\Gamma }$, which ensures that ${\tilde{r}}_{i,l}^{c}$ and ${\tilde{r}}_{i,l}$ share the same $\Delta_{i,l}$. For clarity, all the notations are listed in Table 1. In the following, we aim to estimate the real number $X_{1}$ with known erroneous residues ${\tilde{r}}_{i,l}$ and moduli $m_{l}$.

Since $X_{i}=\left\lfloor X_{i}/\Gamma \right\rfloor \Gamma +r_{i}^{c}$, we recover $X_{i}$ by estimating the folding number $\left\lfloor X_{i}/\Gamma \right\rfloor$ and common residue $r_{i}^{c}$ successively. We adopt the reconstruction framework proposed in [24], which consists of three steps:

(i) Estimate the folding number: Based on the fact that $X_{i}=k_{i}m_{l}+r_{i,l}=k_{i}M_{l}\Gamma +r_{i,l}$, where $k_{i}\in Z$, we have $\left\lfloor X_{i}/\Gamma \right\rfloor =k_{i}M_{l}+\left\lfloor r_{i,l}/\Gamma \right\rfloor =k_{i}M_{l}+\left(r_{i,l}-{\langle r_{i,l}\rangle }_{\Gamma }\right)/\Gamma$. Clearly, ${\langle r_{i,l}\rangle }_{\Gamma }={\langle {\langle X_{i}\rangle }_{M_{l}\Gamma }\rangle }_{\Gamma }={\langle X_{i}\rangle }_{\Gamma }=r_{i}^{c}$. Thus, we have $\left\lfloor X_{i}/\Gamma \right\rfloor =k_{i}M_{l}+\left(r_{i,l}-r_{i}^{c}\right)/\Gamma$. By taking the modulo arithmetic, one can obtain
  (4) $$\left\lfloor \frac{X_{i}}{\Gamma }\right\rfloor \equiv \frac{r_{i,l}-r_{i}^{c}}{\Gamma }\;\mathrm{mod}\;M_{l},$$
  From (4), the folding number $\left\lfloor X_{i}/\Gamma \right\rfloor$ is estimated by the equation below via CRT [24],
  (5) $$q_{i}\equiv \frac{{\tilde{r}}_{i,l}-{\tilde{r}}_{i,l}^{c}}{\Gamma }\;\mathrm{mod}\;M_{l},$$
  where $q_{i}$ denotes the estimation of $\left\lfloor X_{i}/\Gamma \right\rfloor$.
(ii) Estimate the common residues: Calculate $\sum_{l=1}^{L}{\tilde{r}}_{i,l}^{c}/L$ as the estimation of the common residue $r_{i}^{c}$.
(iii) Estimate the number: Based on $X_{i}=\left\lfloor X_{i}/\Gamma \right\rfloor \Gamma +r_{i}^{c}$, $X_{i}$ is reconstructed by
  (6) $$\hat{X_{i}}=q_{i}\Gamma +\frac{\sum_{l=1}^{L}{\tilde{r}}_{i,l}^{c}}{L},$$
  where ${\hat{X}}_{i}$ represents the estimation of $X_{i}$.

### 2.3. Key Issues in Real Waveform

(i) Robustness: Trivially estimating the folding number by (5) may lead to large errors due to the ambiguity of $({\tilde{r}}_{i,l}-{\tilde{r}}_{i,l}^{c})/\Gamma$. In other words, since $r_{i}^{c}\in \left[0,\Gamma \right)$ and $|\Delta_{i,l}|<\Gamma /4$, $({\tilde{r}}_{i,l}-{\tilde{r}}_{i,l}^{c})/\Gamma$ must satisfy one of the three subcases below based on (4) [33],
  (7) $$\begin{array}{c} \frac{{\tilde{r}}_{i,l}-{\tilde{r}}_{i,l}^{c}}{\Gamma }\equiv \left\{\begin{array}{c} \left\lfloor X_{i}/\Gamma \right\rfloor \;\;\;\;\;\;\;\mathrm{mod}\;M_{l},\;\;\mathrm{if}\;\;r_{i}^{c}+\Delta_{i,l}\in \left[0,\Gamma \right)\\ \left\lfloor X_{i}/\Gamma \right\rfloor -1\;\;\mathrm{mod}\;M_{l},\;\;\mathrm{if}\;\;r_{i}^{c}+\Delta_{i,l}\in \left(-\Gamma /4,0\right)\\ \left\lfloor X_{i}/\Gamma \right\rfloor +1\;\;\;\mathrm{mod}\;M_{l},\;\;\mathrm{if}\;\;r_{i}^{c}+\Delta_{i,l}\in \left[\Gamma ,5\Gamma /4\right) \end{array}\right. \end{array}$$
  If $r_{i}^{c}+\Delta_{i,l_{1}}$ and $r_{i}^{c}+\Delta_{i,l_{2}}$ fall into different subcases in (7), where $l_{1},l_{2}\in \left\{1,2,\cdots ,L\right\}$, simply aggregating them via CRT will bring unpredictable reconstruction errors. Thus, we need to unify $({\tilde{r}}_{i,l}-{\tilde{r}}_{i,l}^{c})/\Gamma$ such that all of them fall into one subcase in (7) to ensure robustness. This can be achieved by sorting ${\tilde{r}}_{i,l}^{c}$, where ${\tilde{r}}_{i,l}^{c}={\langle r_{i}^{c}+\Delta_{i,l}\rangle }_{\Gamma }$, in the order such that the corresponding $\Delta_{i,l}$ are in an ascending order for each *i*. However, the above operation is only implementable when $|\Delta_{i,l}|<\Gamma /8$ [34], while it still remains open in the generic setup $|\Delta_{i,l}|<\Gamma /4$.
(ii) Residue Ambiguity: Due to the loss of the correspondence between $X_{i}$ and ${\tilde{r}}_{i,l}$, we cannot cluster ${\tilde{r}}_{i,l}$ corresponding to $X_{i}$ to calculate $q_{i}$ from (5) for each *i*.

## 3. Robust Reconstruction for Frequency Estimation of Single-Tone Real Waveform

This section presents the polynomial-time RCRT-based frequency estimation for a noisy single-tone real waveform. Before proceeding, the following notations are introduced.

We first define a metric to represent the minimum circular distance between ${\tilde{r}}_{i,l_{1}}^{c}$ and ${\tilde{r}}_{i,l_{2}}^{c}$ on the circle of length Γ, i.e.,

(8) $$d\left({\tilde{r}}_{i,l_{1}}^{c},{\tilde{r}}_{i,l_{2}}^{c}\right)=\min_{z}\left|{\tilde{r}}_{i,l_{1}}^{c}-{\tilde{r}}_{i,l_{2}}^{c}+z\Gamma \right|,\;z\in \left\{-1,0,1\right\}.$$

**Figure 1:**
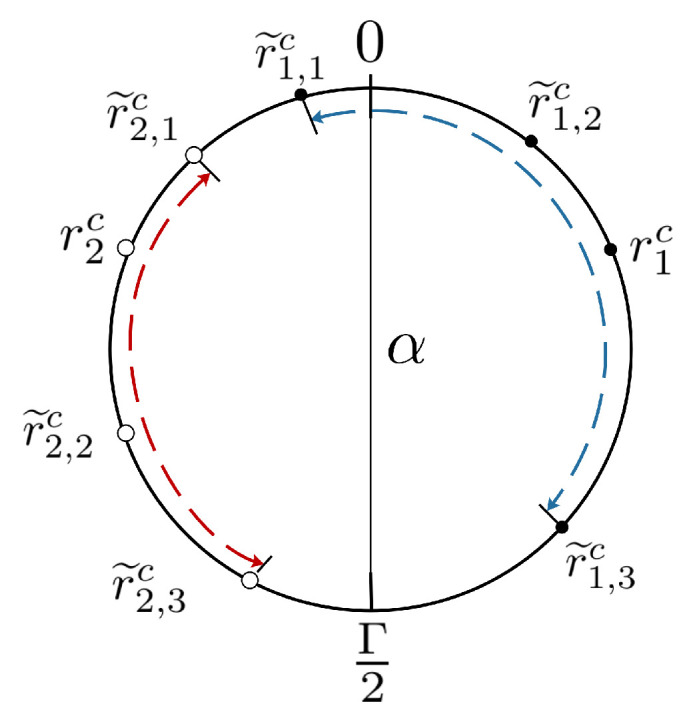
Illustration of the intervals.

For example, if Γ=12, d(1,11)=2. Let $I({\tilde{r}}_{i,l_{1}}^{c},{\tilde{r}}_{i,l_{2}}^{c})$ denote the interval between ${\tilde{r}}_{i,l_{1}}^{c}$ and ${\tilde{r}}_{i,l_{2}}^{c}$ on the circle (such as $I({\tilde{r}}_{1,1}^{c},{\tilde{r}}_{1,3}^{c})$ shown in Figure 1), whose length is $d({\tilde{r}}_{i,l_{1}}^{c},{\tilde{r}}_{i,l_{2}}^{c})$. $\max_{l}I\left({\tilde{r}}_{i,l_{1}}^{c},{\tilde{r}}_{i,l_{2}}^{c}\right)$ represents the interval whose length is maximal. As shown in Figure 1, when i=1, the maximum interval is $I({\tilde{r}}_{1,1}^{c},{\tilde{r}}_{1,3}^{c})$; when i=2, $I({\tilde{r}}_{2,1}^{c},{\tilde{r}}_{2,3}^{c})$ is the maximum one.

### 3.1. The Order of Residues

Now, we consider the first key issue stated in Section 2.3, i.e., sorting ${\tilde{r}}_{i,l}^{c}$ in the order such that the corresponding errors $\Delta_{i,l}$ are in ascending order for each *i*, where ${\tilde{r}}_{i,l}^{c}={\langle r_{i}^{c}+\Delta_{i,l}\rangle }_{\Gamma }$. According to [21], sorting is equivalent to finding a cutting point ξ on the circle of length Γ and stretching it to a real axis. If $\xi \notin \max_{l}I\left({\tilde{r}}_{i,l_{1}}^{c},{\tilde{r}}_{i,l_{2}}^{c}\right)$ for each *i*, the shifted common residues ${\hat{r}}_{i,l}^{c}$ on the real axis are sorted in ascending order of $\Delta_{i,l}$.

**Figure 2:**
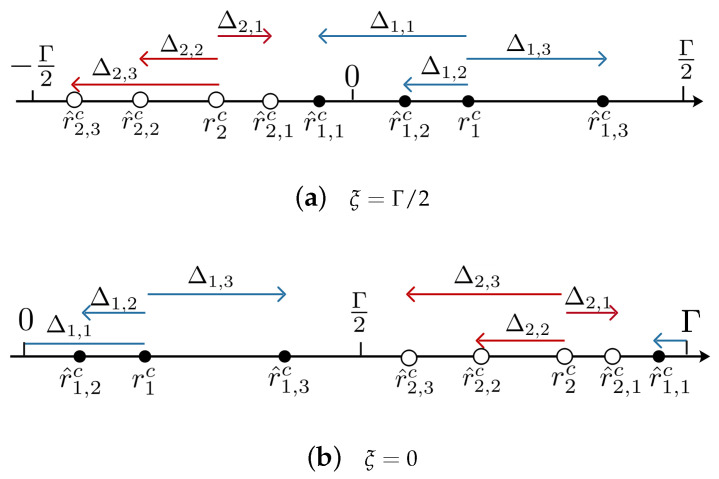
Sketch of the Definition of ${\hat{r}}_{i,l}^{c}$.

For example, in Figure 1, Γ/2 is not in the maximum intervals, i.e., $I({\tilde{r}}_{1,1}^{c},{\tilde{r}}_{1,3}^{c})$ and $I({\tilde{r}}_{2,1}^{c},{\tilde{r}}_{2,3}^{c})$. Then, cutting the circle at Γ/2 leads to ${\hat{r}}_{2,3}^{c}<{\hat{r}}_{2,2}^{c}<{\hat{r}}_{2,1}^{c}$ and ${\hat{r}}_{1,1}^{c}<{\hat{r}}_{1,2}^{c}<{\hat{r}}_{1,3}^{c}$ sorted in ascending order of $\Delta_{i,l}$, i.e., $\Delta_{2,3}<\Delta_{2,2}<0<\Delta_{2,1}$ and $\Delta_{1,1}<\Delta_{1,2}<0<\Delta_{1,3}$ shown in Figure 2a. On the contrary, if we cut the circle at 0, where $0\in I({\tilde{r}}_{1,1}^{c},{\tilde{r}}_{1,3}^{c})$, $\Delta_{1,1}$ breaks the ascending order, as shown in Figure 2b. Here, ${\hat{r}}_{1,2}^{c}<{\hat{r}}_{1,3}^{c}<{\hat{r}}_{1,1}^{c}$, but $\Delta_{1,2},\Delta_{1,3},\Delta_{1,1}$ are in non-ascending order.

However, the key remaining problem is how to find the proper cutting point without the correspondence between ${\tilde{r}}_{i,l}^{c}$ and $X_{i}$, i.e., $\max_{l}I\left({\tilde{r}}_{i,l_{1}}^{c},{\tilde{r}}_{i,l_{2}}^{c}\right)$ is unknown. To this end, ref. [21] sacrifices the error bound to Γ/8. Nonetheless, we reach the error bound Γ/4 by using the symmetry of residues, i.e.,

$$r_{2}^{c}={\langle X_{2}\rangle }_{\Gamma }={\langle -X_{1}\rangle }_{\Gamma }=\Gamma -r_{1}^{c}.$$

That is to say, $r_{1}^{c}$ and $r_{2}^{c}$ are axially symmetric about line α shown in Figure 1. Since $|\Delta_{i,l}|<\Gamma /4$, $d\left({\tilde{r}}_{i,l_{1}}^{c},{\tilde{r}}_{i,l_{2}}^{c}\right)<d\left(r_{i}^{c}-\Gamma /4,r_{i}^{c}+\Gamma /4\right)=\Gamma /2$, i.e., $\max_{l}I\left({\tilde{r}}_{i,l_{1}}^{c},{\tilde{r}}_{i,l_{2}}^{c}\right)$ cannot contain 0 and Γ/2 simultaneously. Based on the symmetry, $\max_{l}I\left({\tilde{r}}_{1,l_{1}}^{c},{\tilde{r}}_{1,l_{2}}^{c}\right)\cup \max_{l}I\left({\tilde{r}}_{2,l_{1}}^{c},{\tilde{r}}_{2,l_{2}}^{c}\right)$ cannot contain both 0 and Γ/2. Thus, either 0 or Γ/2 is the cutting point. To figure out the cutting point, we state Lemma 1, which is proved in Appendix A, that once $0\in \max_{l}I\left({\tilde{r}}_{i,l_{1}}^{c},{\tilde{r}}_{i,l_{2}}^{c}\right)$, $\min_{il}d\left(0,{\tilde{r}}_{i,l}^{c}\right)<\min_{il}d\left(\Gamma /2,{\tilde{r}}_{i,l}^{c}\right)$ holds. Similarly, $\min_{il}d\left(0,{\tilde{r}}_{i,l}^{c}\right)>\min_{il}d\left(\Gamma /2,{\tilde{r}}_{i,l}^{c}\right)$ is true when $\max_{l}I\left({\tilde{r}}_{i,l_{1}}^{c},{\tilde{r}}_{i,l_{2}}^{c}\right)$ contains Γ/2. Thus, the unknown $\max_{l}I\left({\tilde{r}}_{i,l_{1}}^{c},{\tilde{r}}_{i,l_{2}}^{c}\right)$ problem is converted to distance comparison, i.e., $\min_{il}d\left(0,{\tilde{r}}_{i,l}^{c}\right)$ and $\min_{il}d\left(\Gamma /2,{\tilde{r}}_{i,l}^{c}\right)$.

Before giving Lemma 1, we define Operation 1 and 2 corresponding to the cutting point is 0 and Γ/2, respectively.

- Operations 1:
  (9) $${\hat{r}}_{i,l}^{c}={\tilde{r}}_{i,l}^{c}$$
- Operation 2:
  (10) $${\hat{r}}_{i,l}^{c}={\tilde{r}}_{i,l}^{c}\;\mathrm{if}\;{\tilde{r}}_{i,l}^{c}\in \left[0,\frac{\Gamma }{2}\right),\;\mathrm{otherwise}\;{\hat{r}}_{i,l}^{c}={\tilde{r}}_{i,l}^{c}-\Gamma$$

 **Lemma 1.**  *If $\min_{il}d\left(0,{\tilde{r}}_{i,l}^{c}\right)<\min_{il}d\left(\Gamma /2,{\tilde{r}}_{i,l}^{c}\right)$, where 1≤i≤2 and 1≤l≤L, apply Operation 2 on ${\tilde{r}}_{i,l}^{c}$; otherwise, Operation 1. The resultant ${\hat{r}}_{i,l}^{c}$ are sorted in ascending order of $\Delta_{i,l}$ for each i.*

For example, as shown in Figure 1, $\min_{il}d\left(0,{\tilde{r}}_{i,l}^{c}\right)=d\left(0,{\tilde{r}}_{1,1}^{c}\right)<\min_{il}d\left(\Gamma /2,{\tilde{r}}_{i,l}^{c}\right)=d\left(\Gamma /2,{\tilde{r}}_{2,3}^{c}\right)$. Thus, Operation 2 is applied. The resultant ${\hat{r}}_{i,l}^{c}$ are sorted in the order that the corresponding $\Delta_{i,l}$ are in ascending order for each *i*, shown in Figure 2a.

### 3.2. Residue Ambiguity

With ${\hat{r}}_{i,l}^{c}$ sorted in ascending order of $\Delta_{i,l}$, $({\tilde{r}}_{i,l}-{\hat{r}}_{i,l}^{c})/\Gamma$ fall into one subcase in (7) [24]. Now, we discuss the second key issue, i.e., residue ambiguity. If we can divide ${\tilde{r}}_{i,l}$ into two sets corresponding to $X_{1}$ and $X_{2}$, respectively, the folding number $\left\lfloor X_{i}/\Gamma \right\rfloor$ can be estimated based on (5) [34]:

(11) $$q_{i}\equiv \frac{{\tilde{r}}_{i,l}-{\hat{r}}_{i,l}^{c}}{\Gamma }\;\mathrm{mod}\;M_{l}.$$

However, the correspondence between ${\tilde{r}}_{i,l}$ and $X_{i}$ is unknown. To determine the correspondence, Li et al. proposed a scheme for positive numbers, which cannot be directly applied to the real waveform since $X_{2}<0$ [23]. To solve this issue, we form a quadratic equation by the prior condition $X_{2}=-X_{1}$. First, we consider the two residues $\left\{\left({\tilde{r}}_{1,l}-{\hat{r}}_{1,l}^{c}\right)/\Gamma ,\left({\tilde{r}}_{2,l}-{\hat{r}}_{2,l}^{c}\right)/\Gamma \right\}$ as a pair for each *l*. By multiplying each pair, we can reconstruct $q_{1}q_{2}$ via CRT based on (11) [22], i.e.,

(12) $$q_{1}q_{2}\equiv \frac{{\tilde{r}}_{1,l}-{\hat{r}}_{1,l}^{c}}{\Gamma }\times \frac{{\tilde{r}}_{2,l}-{\hat{r}}_{2,l}^{c}}{\Gamma }\;\mathrm{mod}\;M_{l}.$$

Then, with $X_{2}=-X_{1}$, it can be proved that either $q_{2}=-q_{1}$ or $q_{2}=-q_{1}-1$ holds, which is stated in Lemma 2 and proved in Appendix B. Hence, we can form a quadratic equation in one unknown by replacing $q_{2}$ with $-q_{1}$ or $-q_{1}-1$ in (12) based on Lemma 2. In a nutshell, the residue ambiguity is addressed by solving one of the two quadratic equations below via CRT, corresponding to $q_{2}=-q_{1}$ and $q_{2}=-q_{1}-1$, respectively.

(13) $$q_{1}^{2}\equiv M_{l}-\frac{{\tilde{r}}_{1,l}-{\hat{r}}_{1,l}^{c}}{\Gamma }\times \frac{{\tilde{r}}_{2,l}-{\hat{r}}_{2,l}^{c}}{\Gamma }\;\mathrm{mod}\;M_{l}$$

(14) $$q_{1}^{2}+q_{1}\equiv M_{l}-\frac{{\tilde{r}}_{1,l}-{\hat{r}}_{1,l}^{c}}{\Gamma }\times \frac{{\tilde{r}}_{2,l}-{\hat{r}}_{2,l}^{c}}{\Gamma }\;\mathrm{mod}\;M_{l}$$

 **Lemma 2.**  *If $|\Delta_{i,l}|<\Gamma /4$, $\left\{q_{1},q_{2}\right\}$ must fall into one of the following two cases:*

- *$q_{2}=-q_{1}-1$, when Operation 1 is the appropriate operation and performed on ${\tilde{r}}_{i,l}^{c}$, where $q_{1}=\left\lfloor X_{1}/\Gamma \right\rfloor$.*
- *$q_{2}=-q_{1}$, when Operation 2 is the appropriate operation and performed on ${\tilde{r}}_{i,l}^{c}$. If $r_{1}^{c}\in \left[0,\Gamma /2\right)$, $q_{1}=\left\lfloor X_{1}/\Gamma \right\rfloor$. Otherwise, $q_{1}=\left\lfloor X_{1}/\Gamma \right\rfloor +1$.*

### 3.3. Reconstruction Scheme

With identified $q_{1}$, we consider the last two steps mentioned in Section 2.2. Clearly, ${\hat{r}}_{1,l}^{c}$ can be distinguished from (11) since $q_{1}$ is determined. Thus, $X_{1}$ is estimated based on Section 2.2:

(15) $${\hat{X}}_{1}=q_{1}\Gamma +\frac{\sum_{l=1}^{L}{\hat{r}}_{1,l}^{c}}{L}.$$

With the above understanding, we state the final conclusion, i.e., Theorem 1, that reconstruction error is bounded by Γ/4, where the proof is in Appendix C.

 **Theorem 1.**  *If $X_{1}\in \left[0,\left\lfloor \sqrt{M}\right\rfloor \Gamma -\Gamma /2\right)$ and $|\Delta_{i,l}|<\Gamma /4$, $|{\hat{X}}_{1}-X_{1}|<\Gamma /4$ holds, where $M=\prod_{l=1}^{L}M_{l}$.*

For step 4 of Algorithm 1, the time complexity of solving the Equation (13) or (14) via CRT is O(1). Since we need to process at most 2L${\tilde{r}}_{i,l}^{c}$ or ${\hat{r}}_{i,l}^{c}$ in each step, the time complexity of Algorithm 1 is O(L).
**Algorithm 1** Robust frequency estimation for the single-tone real waveform.**Input**: Moduli: $\left\{m_{l}|m_{l}=M_{l}\Gamma ,l=1,2,\cdots ,L\right\}$.Erroneous residue sets: $S_{l}=\left\{{\tilde{r}}_{1,l},{\tilde{r}}_{2,l}\right\}$, l=1,2,⋯,L.   1:Calculate the erroneous common residues ${\tilde{r}}_{i,l}^{c}={\langle {\tilde{r}}_{i,l}\rangle }_{\Gamma }$.   2:Calculate $\min_{il}d\left(0,{\tilde{r}}_{i,l}^{c}\right)$ and $\min_{il}d\left(\Gamma /2,{\tilde{r}}_{i,l}^{c}\right)$ from (8).   3:If $\min_{il}d\left(0,{\tilde{r}}_{i,l}^{c}\right)<\min_{il}d\left(\Gamma /2,{\tilde{r}}_{i,l}^{c}\right)$, perform (10) on ${\tilde{r}}_{i,l}^{c}$ to obtain ${\hat{r}}_{i,l}^{c}$. Otherwise, perform (9).   4:If (9) is applied, solve the Equation (14) via CRT to obtain $q_{1}$. Otherwise, solve the Equation (13).   5:Cluster the shifted common residues ${\hat{r}}_{i,l}^{c}$ satisfying (11).   6:Calculate ${\hat{X}}_{1}$ according to (15).**Output**: ${\hat{X}}_{1}$

 **Example 1.**  *Operation 1 is applied. The moduli are $m_{l}=M_{l}\Gamma \in \left\{3\times 10,5\times 10,7\times 10\right\}$, where the greatest common divisor Γ=10 and $|\Delta_{i,l}|<\Gamma /4$. If $X_{1}=94$ and $X_{2}=-94$, we assume the erroneous residue sets are $S_{1}=\left\{2,25\right\}$, $S_{2}=\left\{44,7\right\}$, and $S_{3}=\left\{23,48\right\}$. Thus, the erroneous common residues are $\left\{{\tilde{r}}_{1,1}^{c},{\tilde{r}}_{2,1}^{c}\right\}=\left\{2,5\right\}$, $\left\{{\tilde{r}}_{1,2}^{c},{\tilde{r}}_{2,2}^{c}\right\}=\left\{4,7\right\}$, and $\left\{{\tilde{r}}_{1,3}^{c},{\tilde{r}}_{2,3}^{c}\right\}=\left\{3,8\right\}$. Clearly, $\min_{il}d\left(0,{\tilde{r}}_{i,l}^{c}\right)=2>\min_{il}d\left(5,{\tilde{r}}_{i,l}^{c}\right)=0$, so Operation 1 is performed, i.e., ${\hat{r}}_{i,l}^{c}={\tilde{r}}_{i,l}^{c}$. According to (14), we obtain: (1). $q_{1}^{2}+q_{1}\equiv 0\;\mathrm{mod}\;3$; (2). $q_{1}^{2}+q_{1}\equiv 0\;\mathrm{mod}\;5$; (3). $q_{1}^{2}+q_{1}\equiv 6\;\mathrm{mod}\;7$. One can obtain $q_{1}^{2}+q_{1}=90$ via CRT, which leads to $q_{1}=9$. From (11), the shifted common residues ${\hat{r}}_{i,l}^{c}$ of $q_{1}$ are {2,4,3}. So ${\hat{X}}_{1}=9\times 10+\left(2+4+3\right)/3=93$.*

 **Example 2.**  *Operation 2 is applied. Likewise, the moduli are $m_{l}=M_{l}\Gamma \in \left\{3\times 10,5\times 10,7\times 10\right\}$. If $X_{1}=81$ and $X_{2}=-81$, the erroneous residue sets are assumed as $S_{1}=\left\{20,11\right\}$, $S_{2}=\left\{29,18\right\}$, and $S_{3}=\left\{12,57\right\}$. Correspondingly, the erroneous common residues are $\left\{{\tilde{r}}_{1,1}^{c},{\tilde{r}}_{2,1}^{c}\right\}=\left\{0,1\right\}$, $\left\{{\tilde{r}}_{1,2}^{c},{\tilde{r}}_{2,2}^{c}\right\}=\left\{9,8\right\}$, and $\left\{{\tilde{r}}_{1,3}^{c},{\tilde{r}}_{2,3}^{c}\right\}=\left\{2,7\right\}$. Clearly, $\min_{il}d\left(0,{\tilde{r}}_{i,l}^{c}\right)=0<\min_{il}d\left(5,{\tilde{r}}_{i,l}^{c}\right)=2$, so Operation 2 is applied on ${\tilde{r}}_{i,l}^{c}$. Thus, we obtain the shifted residues based on (10): $\left\{{\hat{r}}_{1,1}^{c},{\hat{r}}_{2,1}^{c}\right\}=\left\{0,1\right\}$, $\left\{{\hat{r}}_{1,2}^{c},{\hat{r}}_{2,2}^{c}\right\}=\left\{-1,-2\right\}$, and $\left\{{\hat{r}}_{1,3}^{c},{\hat{r}}_{2,3}^{c}\right\}=\left\{2,-3\right\}$. According to (13), one can derive that: (1). $q_{1}^{2}\equiv 1\;\mathrm{mod}\;3$; (2). $q_{1}^{2}\equiv 4\;\mathrm{mod}\;5$; (3). $q_{1}^{2}\equiv 1\;\mathrm{mod}\;7$. Based on CRT, we have $q_{1}^{2}=64$, resulting in $q_{1}=8$. With determined $q_{1}$, we continue to figure out the corresponding shifted common residues based on (11), i.e., {0,−1,2}. As a result, ${\hat{X}}_{1}=8\times 10+\left(0-1+2\right)/3=80.33$.*

## 4. Simulation Results

In this section, we first present some simulations to verify our proposed theory. Then the simulation results are shown to demonstrate the performance of the proposed method compared with that of the robust generalized CRT [23] and searching−based algorithm [29].

In the following, we first consider the estimation error versus the error upper bound for our proposed theory, i.e., Theorem 1. To begin with, the simulation setup is given as follows. The moduli are $m_{l}=\left\{11\times 80,13\times 80,17\times 80\right\}$, where the greatest common factor Γ=80 and the maximal error level τ∈{1,2,⋯,25}. Based on Theorem 1, τ needs to be bounded by Γ/4=20 to ensure robustness.

For a trial, one unknown real number *X* is chosen randomly, which belongs to the dynamic range [0,3880), where the negative duplicate is −X. Moreover, 10,000 trials are implemented for each τ.

**Figure 3:**
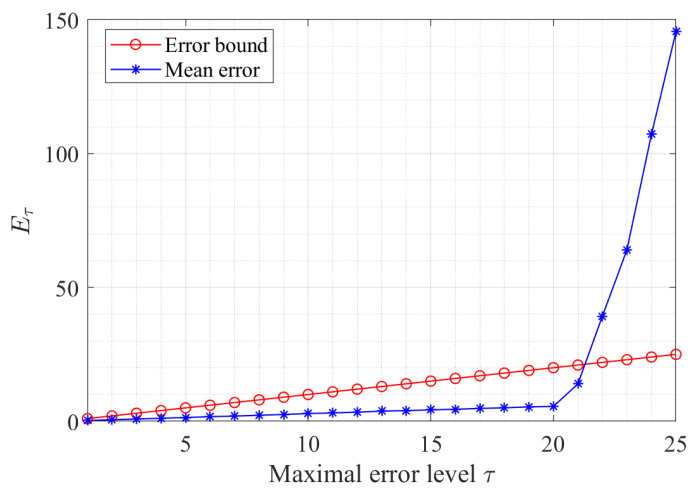
Estimation errors versus the maximal error level.

Figure 3 shows the mean absolute error $E_{\tau }$ between the estimate $\hat{X}$ and the true number *X* for each error bound. The mean absolute error $E_{\tau }$ is defined as below,

(16) $$E_{\tau }=E_{trials}\left(\right|\hat{X}-X\left|\right),$$

where $E_{trials}$ denotes the mean of all the trials, $\hat{X}$ and *X* are the estimate and true number in a trial, respectively. Clearly, $E_{\tau }$ is less than τ when τ≤Γ/4=20, which matches well with our conclusion. Once τ exceeds the error bound, the reconstruction error increases rapidly.

**Figure 4:**
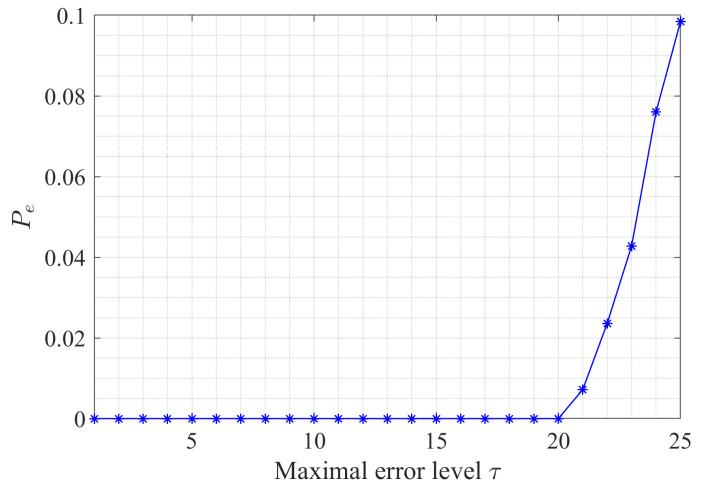
Probability $P_{e}$ versus the maximal error level.

In Figure 4, we present the curve of the probability of failure $P_{e}$ versus the error bound τ, where

(17) $$P_{e}=P(|\hat{X}-X\left|>\tau \right).$$

One can see that when τ≤Γ/4, the probability of failure is zero while non−zero when τ exceeds the bound. In a word, if $\tau <\frac{\Gamma }{4}$, the reconstruction error is linearly bounded by τ, the probability of which is 1.

Next, we compare the performance of the proposed algorithm with that of the robust generalized CRT for two numbers in [23] and the searching−based algorithm in [29]. We consider the real sinusoidal waveform case and select L=3 sampling rates (moduli) in a form m[1:L]=Γ×{11,13,17}, which share a greatest common factor Γ. We test different sampling rates where Γ={40,80}. The unknown frequency *X* is randomly selected from the range [0,48.5×Γ). Each noise $\Delta_{i,l}$ is assumed to be some independent uniform noise within (−τ,τ), where τ varies from 1 to 25.

We repeat 5000 trials for each selection of Γ and τ. On the on hand, the root mean square error (RMSE) is investigated, where

(18) $$\mathrm{RMSE}={\left\{E{\left(\hat{X}-X\right)}^{2}\right\}}^{1/2}.$$

**Figure 5:**
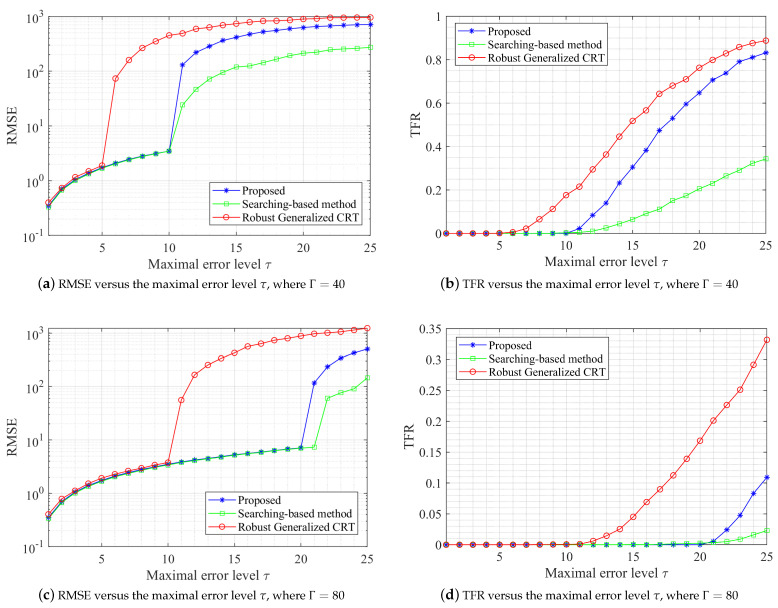
Performance simulation comparison among searching-based method [29], robust generalized CRT [23] and proposed RCRT.

Figure 5a,c show that our method outperforms the best known robust generalized CRT, where the maximal error tolerance is improved from Γ/8 to Γ/4. Morever, our method performs as well as the best searching−based method when the maximal error level is less than Γ/4.

On the other hand, we compare the test fail rate (TFR). We say that the test fails when

(19) $$\mathrm{TFR}=P(|\hat{X}-X|>\Gamma /4).$$

As shown in Figure 5b,d, if τ≤Γ/4, the estimation error is bounded by Γ/4, the probability of which is one. Once the maximal error level exceeds Γ/4, the reconstruction error is almost unpredictable. In a word, our method outperforms the robust generalized CRT while slightly worse than the searching−based algorithm when τ>Γ/4. However, it’s worth pointing out that our method provides a closed−form solution that cannot be realized by the searching−based method. Then, we consider the real running time consumption, where the computing equipment is Lenovo xiaoxin Pro 13. The real running time of our method that runs for 125,000 times is about 7.96 s, while the robust generalized CRT proposed in [23] requires about 86.05 s since the algorithm involves a lot of loops. The searching-based method proposed in [29] sightly outperforms our method, which only needs about 5.75 s.

## 5. Discussion

The experiment results in Figure 5 suggest a clear improvement in the error bound from Γ/8 to Γ/4 compared with the method proposed in [23]. The reason why we can improve the error bound is that we fully utilized the prior knowledge of the real waveform, i.e., symmetry. For the real waveform, the real peak and the corresponding spurious peak are symmetric at about 0 points in the spectrum. Thus, the frequency determination from undersampled single-tone real waveform can be formulated as RCRT for two numbers $\left\{X_{1},X_{2}\right\}$, where these two numbers are in a form $X_{1}=-X_{2}$. Based on this symmetry, the corresponding error-free common residues $\left\{r_{1}^{c},r_{2}^{c}\right\}$ are symmetric on the circle of length Γ. The geometric property of symmetry ensures that even if the error bound is improved to Γ/4, we can still shift the erroneous common residues correctly to obtain a robust reconstruction. In addition, our algorithm is also highly efficient according to the real running time and the theoretical analysis. We use the prior condition of the real waveform to form a quadratic equation in one unknown to determine the folding numbers, which realizes the high efficiency of the algorithm.

In summary, our proposed method provides a feasible solution for the frequency determination from the undersampled single-tone real waveform. In addition, we complete the study of CRT-based frequency determination from undersampled waveform, which shows that the optimal error tolerance bound can be achieved in the real waveform case. The limitation of our proposed method is that since it is based on the prior knowledge of the real waveform and the prior condition is invalid; it cannot handle the complex waveform. In addition, this algorithm cannot deal with the case of multiple frequency estimation from undersampled real waveforms. We will investigate these problems in our future studies.

## 6. Conclusions

We proposed the first polynomial-time RCRT-based frequency estimation for a noisy single-tone real waveform, which matches the optimal error bound. The proposed method can be applied in SAR imaging of moving targets or sensor networks where the sampling rate may be lower than the Nyquist rate of the input signal. The time complexity of the proposed method is linear to the number of samplers. Moreover, the proposed method can estimate the frequency from the real waveform by sub-Nyquist rates, which reduces the cost and system size, especially in sensor networks that require noticeable sensors. We believe the method can be further extended to the multiple frequencies case.

## Figures and Tables

**Table 1:** List of Notations.

Notations	Explanation
$M_{l}$	Co-prime moduli
$m_{l}$	Moduli selected
$X_{i}$	Number to be recovered
$\hat{X_{i}}$	Estimation of $X_{i}$
$\left\lfloor X_{i}/\Gamma \right\rfloor$	The folding number of $X_{i}$
$q_{i}$	Estimation of $\left\lfloor X_{i}/\Gamma \right\rfloor$
${\tilde{r}}_{i,l}={\langle X_{i}+\Delta_{i,l}\rangle }_{m_{l}}$	Erroneous residue of $X_{i}$ modulo $m_{l}$
$r_{i}^{c}={\langle X_{i}\rangle }_{\Gamma }$	Common residue of $X_{i}$
${\tilde{r}}_{i,l}^{c}={\langle r_{i}^{c}+\Delta_{i,l}\rangle }_{\Gamma }$	Erroneous common residue of $X_{i}$
${\hat{r}}_{i,l}^{c}$	Shifted common residue of $X_{i}$
$d({\tilde{r}}_{i,l_{1}}^{c},{\tilde{r}}_{i,l_{2}}^{c})$	Minimum circular distance between ${\tilde{r}}_{i,l_{1}}^{c}$ and ${\tilde{r}}_{i,l_{2}}^{c}$ on the circle of length Γ
$I({\tilde{r}}_{i,l_{1}}^{c},{\tilde{r}}_{i,l_{2}}^{c})$	Interval between ${\tilde{r}}_{i,l_{1}}^{c}$ and ${\tilde{r}}_{i,l_{2}}^{c}$

## Data Availability

Not applicable.

## Appendix A. Proof of Lemma 1

 **Proof.** For brevity, we only consider the case when $r_{1}^{c}\leq r_{2}^{c}$, i.e., $r_{1}^{c}$ is on the right half of the circle Γ. For the case $r_{1}^{c}\geq r_{2}^{c}$, which is obviously symmetric with $r_{1}^{c}\leq r_{2}^{c}$, one can obtain the same conclusion based on the following idea. Since $\max_{l}I\left({\tilde{r}}_{1,l_{1}}^{c},{\tilde{r}}_{1,l_{2}}^{c}\right)\cup \max_{l}I\left({\tilde{r}}_{2,l_{1}}^{c},{\tilde{r}}_{2,l_{2}}^{c}\right)$ can not contain 0 and Γ/2 simultaneously, the intervals must satisfy one of the three cases:

(1) $0\in \max_{l}I\left({\tilde{r}}_{i,l_{1}}^{c},{\tilde{r}}_{i,l_{2}}^{c}\right)$
(2) $\Gamma /2\in \max_{l}I\left({\tilde{r}}_{i,l_{1}}^{c},{\tilde{r}}_{i,l_{2}}^{c}\right)$
(3) $\max_{l}I\left({\tilde{r}}_{i,l_{1}}^{c},{\tilde{r}}_{i,l_{2}}^{c}\right)$ contains neither 0 nor Γ/2

Case (1): $0\in \max_{l}I\left({\tilde{r}}_{1,l_{1}}^{c},{\tilde{r}}_{1,l_{2}}^{c}\right)$ or $\max_{l}I\left({\tilde{r}}_{2,l_{1}}^{c},{\tilde{r}}_{2,l_{2}}^{c}\right)$. If $0\in \max_{l}I\left({\tilde{r}}_{1,l_{1}}^{c},{\tilde{r}}_{1,l_{2}}^{c}\right)$, there must exist at least one ${\tilde{r}}_{1,k}^{c}$ to the left of the point 0, and another ${\tilde{r}}_{1,l}^{c}$ to the right of the point 0, where k,l∈{1,2,⋯,L}. Since $r_{1}^{c}$ is on the right half of the circle Γ while ${\tilde{r}}_{1,k}^{c}={\langle r_{1}^{c}+\Delta_{1,k}\rangle }_{\Gamma }$ is to the left of the point 0 and $|\Delta_{1,k}|<\Gamma /4$, we have $r_{1}^{c}\in \left[0,\Gamma /4\right)$. Based on the fact that $r_{2}^{c}=\Gamma -r_{1}^{c}$, $r_{2}^{c}\in \left(3\Gamma /4,\Gamma \right]$. The same conclusion can be derived if $0\in \max_{l}I\left({\tilde{r}}_{2,l_{1}}^{c},{\tilde{r}}_{2,l_{2}}^{c}\right)$. Since $r_{1}^{c}\in \left[0,\Gamma /4\right)$ and ${\tilde{r}}_{1,k}^{c}$ is to the left of the point 0, we obtain

(A1) $$\min_{il}d\left(0,{\tilde{r}}_{i,l}^{c}\right)\leq d\left(0,{\tilde{r}}_{1,k}^{c}\right)<d\left(0,r_{1}^{c}-\frac{\Gamma }{4}\right)=\frac{\Gamma }{4}-r_{1}^{c}.$$

Now, we calculate $\min_{il}d\left(\frac{\Gamma }{2},{\tilde{r}}_{i,l}^{c}\right)$. When i=1, since $r_{1}^{c}\in \left[0,\Gamma /4\right)$ and $|\Delta_{i,l}|<\Gamma /4$, it’s clear that $\min_{l}d\left(\frac{\Gamma }{2},{\tilde{r}}_{1,l}^{c}\right)>d\left(\frac{\Gamma }{2},r_{1}^{c}+\frac{\Gamma }{4}\right)=\frac{\Gamma }{4}-r_{1}^{c}$. When i=2, replacing $r_{2}^{c}$ with $\Gamma -r_{1}^{c}$, we have $\min_{l}d\left(\frac{\Gamma }{2},{\tilde{r}}_{2,l}^{c}\right)>d\left(\frac{\Gamma }{2},r_{2}^{c}-\frac{\Gamma }{4}\right)=\frac{\Gamma }{4}-r_{1}^{c}$. In conclusion, when $0\in \max_{l}I\left({\tilde{r}}_{i,l_{1}}^{c},{\tilde{r}}_{i,l_{2}}^{c}\right)$, we have

(A2) $$\min_{il}d\left(0,{\tilde{r}}_{i,l}^{c}\right)<\frac{\Gamma }{4}-r_{1}^{c}<\min_{il}d\left(\frac{\Gamma }{2},{\tilde{r}}_{i,l}^{c}\right).$$

Since Cases (1) and (2) cannot hold simultaneously, we have $\frac{\Gamma }{2}\notin \max_{l}I\left({\tilde{r}}_{i,l_{1}}^{c},{\tilde{r}}_{i,l_{2}}^{c}\right)$ for each *i*. Thus, cutting the circle at Γ/2, i.e., applying Operation 2 on ${\tilde{r}}_{i,l}^{c}$ leads to ${\hat{r}}_{i,l}^{c}$ sorted in ascending order of $\Delta_{i,l}$. Case (2): $\Gamma /2\in \max_{l}I\left({\tilde{r}}_{1,l_{1}}^{c},{\tilde{r}}_{1,l_{2}}^{c}\right)$ or $\max_{l}I\left({\tilde{r}}_{2,l_{1}}^{c},{\tilde{r}}_{2,l_{2}}^{c}\right)$. Case (2) is obviously symmetric with case (1) on the circle of length Γ. The conclusion can be derived based on the same idea. If $\Gamma /2\in \max_{l}I\left({\tilde{r}}_{1,l_{1}}^{c},{\tilde{r}}_{1,l_{2}}^{c}\right)$, there must exist at least one ${\tilde{r}}_{1,k}^{c}$ to the left of the point Γ/2, and another ${\tilde{r}}_{1,l}^{c}$ to the right of the point Γ/2. Likewise, since ${\tilde{r}}_{1,k}^{c}={\langle r_{1}^{c}+\Delta_{1,k}\rangle }_{\Gamma }$ is to the left of the point Γ/2 while $r_{1}^{c}$ is on the right half and $|\Delta_{1,k}|<\Gamma /4$, we have $r_{1}^{c}\in \left(\Gamma /4,\Gamma /2\right]$ and $r_{2}^{c}\in \left[\Gamma /2,3\Gamma /4\right)$. The same conclusion can be derived if $\Gamma /2\in \max_{l}I\left({\tilde{r}}_{2,l_{1}}^{c},{\tilde{r}}_{2,l_{2}}^{c}\right)$. Since $r_{1}^{c}\in \left(\Gamma /4,\Gamma /2\right]$ and ${\tilde{r}}_{1,k}^{c}$ is to the left of the point Γ/2, we obtain

(A3) $$\min_{il}d\left(\frac{\Gamma }{2},{\tilde{r}}_{i,l}^{c}\right)\leq d\left(\frac{\Gamma }{2},{\tilde{r}}_{1,k}^{c}\right)<d\left(\frac{\Gamma }{2},r_{1}^{c}+\frac{\Gamma }{4}\right)=r_{1}^{c}-\frac{\Gamma }{4}.$$

Then, we discuss $\min_{il}d\left(0,{\tilde{r}}_{i,l}^{c}\right)$. When i=1, since $r_{1}^{c}\in \left(\Gamma /4,\Gamma /2\right]$ and $|\Delta_{i,l}|<\Gamma /4$, one can obtain $\min_{l}d\left(0,{\tilde{r}}_{1,l}^{c}\right)>d\left(0,r_{1}^{c}-\frac{\Gamma }{4}\right)=r_{1}^{c}-\frac{\Gamma }{4}$. When i=2, replacing $r_{2}^{c}$ with $\Gamma -r_{1}^{c}$, we have $\min_{l}d\left(0,{\tilde{r}}_{2,l}^{c}\right)>d\left(0,r_{2}^{c}+\frac{\Gamma }{4}\right)=r_{1}^{c}-\frac{\Gamma }{4}$. Based on the discussion above, when $\Gamma /2\in \max_{l}I\left({\tilde{r}}_{i,l_{1}}^{c},{\tilde{r}}_{i,l_{2}}^{c}\right)$, we have

(A4) $$\min_{il}d\left(\frac{\Gamma }{2},{\tilde{r}}_{i,l}^{c}\right)<r_{1}^{c}-\frac{\Gamma }{4}<\min_{il}d\left(0,{\tilde{r}}_{i,l}^{c}\right).$$

Since Cases (1) and (2) cannot hold simultaneously, we have $0\notin \max_{l}I\left({\tilde{r}}_{i,l_{1}}^{c},{\tilde{r}}_{i,l_{2}}^{c}\right)$ for each *i*. Thus, cutting the circle at 0, i.e., applying Operation 1 on ${\tilde{r}}_{i,l}^{c}$ leads to ${\hat{r}}_{i,l}^{c}$ sorted in ascending order of $\Delta_{i,l}$. Case (3): Since neither 0 nor $\frac{\Gamma }{2}$ is covered by $\max_{l}I\left({\tilde{r}}_{i,l_{1}}^{c},{\tilde{r}}_{i,l_{2}}^{c}\right)$ for each *i*, we can implement either of the two operations on ${\tilde{r}}_{i,l}^{c}$. Q.E.D. □

## Appendix B. Proof of Lemma 2

 **Proof.** The proof has two parts to handle the following two cases, respectively.

(1) $\min d\left(0,{\tilde{r}}_{i,l}^{c}\right)\geq \min d\left(\frac{\Gamma }{2},{\tilde{r}}_{i,l}^{c}\right)$
(2) $\min d\left(0,{\tilde{r}}_{i,l}^{c}\right)<\min d\left(\frac{\Gamma }{2},{\tilde{r}}_{i,l}^{c}\right)$

Case (1): Since Operation 1 is the appropriate operation based on Lemma 1, we have ${\hat{r}}_{i,l}^{c}={\tilde{r}}_{i,l}^{c}$ based on (9). Therefore, replacing ${\hat{r}}_{i,l}^{c}$ with ${\tilde{r}}_{i,l}^{c}$, (11) is equal to

(A5) $$q_{i}\equiv \frac{{\tilde{r}}_{i,l}-{\tilde{r}}_{i,l}^{c}}{\Gamma }\;\mathrm{mod}\;M_{l}.$$

Then, we discuss $r_{i}^{c}+\Delta_{i,l}$, which determines $q_{i}$ in (A5). With $r_{i}^{c}\in \left[0,\Gamma \right)$ and $|\Delta_{i,l}|<\frac{\Gamma }{4}$, $r_{i}^{c}+\Delta_{i,l}$ must fall into one of the three subcases:

(a) $r_{i}^{c}+\Delta_{i,l}\in \left(0,\Gamma \right)$
(b) $r_{i}^{c}+\Delta_{i,l}\in \left(-\frac{\Gamma }{4},0\right)$
(c) $r_{i}^{c}+\Delta_{i,l}\in \left(\Gamma ,\frac{5\Gamma }{4}\right)$

If $r_{i}^{c}+\Delta_{i,l}$ satisfy subcase (b), it leads to $\min_{il}d\left(0,{\tilde{r}}_{i,l}^{c}\right)<\frac{\Gamma }{4}<\min_{il}d\left(\frac{\Gamma }{2},{\tilde{r}}_{i,l}^{c}\right)$, a contradiction to $\min_{il}d\left(0,{\tilde{r}}_{i,l}^{c}\right)\geq \min_{il}d\left(\frac{\Gamma }{2},{\tilde{r}}_{i,l}^{c}\right)$. Similarly, if $r_{i}^{c}+\Delta_{i,l}$ satisfy subcase (c), we have $\min_{il}d\left(0,{\tilde{r}}_{i,l}^{c}\right)<\frac{\Gamma }{4}<\min_{il}d\left(\frac{\Gamma }{2},{\tilde{r}}_{i,l}^{c}\right)$, a contradiction to $\min_{il}d\left(0,{\tilde{r}}_{i,l}^{c}\right)\geq \min_{il}d\left(\frac{\Gamma }{2},{\tilde{r}}_{i,l}^{c}\right)$. Consequently, only subcase (a), i.e., $r_{i}^{c}+\Delta_{i,l}\in \left(0,\Gamma \right)$ holds, leading to $\frac{{\tilde{r}}_{i,l}-{\tilde{r}}_{i,l}^{c}}{\Gamma }$ satisfying subcase (1) in (7). Thus, $\left(\frac{{\tilde{r}}_{1,l}-{\tilde{r}}_{1,l}^{c}}{\Gamma },\frac{{\tilde{r}}_{2,l}-{\tilde{r}}_{2,l}^{c}}{\Gamma }\right)$ are residues of $\left(\left\lfloor \frac{X_{1}}{\Gamma }\right\rfloor ,\left\lfloor \frac{X_{2}}{\Gamma }\right\rfloor \right)$ modulo $M_{l}$, where $\left\lfloor \frac{X_{2}}{\Gamma }\right\rfloor =\left\lfloor \frac{-X_{1}}{\Gamma }\right\rfloor =-\left\lfloor \frac{X_{1}}{\Gamma }\right\rfloor -1$. Therefore, when Operation 1 is the appropriate operation, $q_{2}=-q_{1}-1$ and $q_{1}=\left\lfloor \frac{X_{1}}{\Gamma }\right\rfloor$. Case (2): In this case, Operation 2 is the appropriate operation based on Lemma 1. Then, based on (10), ${\hat{r}}_{i,l}^{c}={\tilde{r}}_{i,l}^{c}$ when ${\tilde{r}}_{i,l}^{c}\in \left[0,\Gamma /2\right)$; otherwise, ${\hat{r}}_{i,l}^{c}={\tilde{r}}_{i,l}^{c}-\Gamma$. Thus, replacing ${\hat{r}}_{i,l}^{c}$ with ${\tilde{r}}_{i,l}^{c}$, (11) is equal to

(A6) $$\begin{array}{c} q_{i}\equiv \left\{\begin{array}{c} \frac{{\tilde{r}}_{i,l}-{\tilde{r}}_{i,l}^{c}}{\Gamma }\;\mathrm{mod}\;M_{l},\;\;\mathrm{if}\;\;{\tilde{r}}_{i}^{c}\in \left[0,\Gamma /2\right)\\ \frac{{\tilde{r}}_{i,l}-{\tilde{r}}_{i,l}^{c}}{\Gamma }+1\;\mathrm{mod}\;M_{l},\;\;\mathrm{otherwise} \end{array}\right. \end{array}$$

Likewise, we discuss $r_{i}^{c}+\Delta_{i,l}$ in the following to figure out $q_{i}$, where $r_{i}^{c}+\Delta_{i,l}$ must fall into one of the five subcases:

(a) $r_{i}^{c}+\Delta_{i,l}\in \left(\frac{\Gamma }{4},\frac{3\Gamma }{4}\right)$
(b) $r_{i}^{c}+\Delta_{i,l}\in \left[0,\frac{\Gamma }{4}\right]$
(c) $r_{i}^{c}+\Delta_{i,l}\in \left[\frac{3\Gamma }{4},\Gamma \right)$
(d) $r_{i}^{c}+\Delta_{i,l}\in \left(-\frac{\Gamma }{4},0\right)$
(e) $r_{i}^{c}+\Delta_{i,l}\in \left(\Gamma ,\frac{5\Gamma }{4}\right)$

If $r_{i}^{c}+\Delta_{i,l}$ satisfies subcase a), we have $\min_{il}d\left(0,{\tilde{r}}_{i,l}^{c}\right)>\frac{\Gamma }{4}>\min_{il}d\left(\frac{\Gamma }{2},{\tilde{r}}_{i,l}^{c}\right)$, a contradiction to $\min_{il}d\left(0,{\tilde{r}}_{i,l}^{c}\right)\leq \min_{il}d\left(\frac{\Gamma }{2},{\tilde{r}}_{i,l}^{c}\right)$. Consequently, subcase (a) cannot happen. Next, we set out to figure out $q_{i}$ corresponding to subcases (b), (c), (d), and (e). Subcase (b): Since $r_{i}^{c}+\Delta_{i,l}\in \left[0,\frac{\Gamma }{4}\right]$, we have ${\tilde{r}}_{i,l}^{c}={\langle r_{i}^{c}+\Delta_{i,l}\rangle }_{\Gamma }\in \left[0,\frac{\Gamma }{4}\right]$. Thus, based on (A6), we have $q_{i}\equiv \frac{{\tilde{r}}_{i,l}-{\tilde{r}}_{i,l}^{c}}{\Gamma }$. Moreover, it is clear that $\frac{{\tilde{r}}_{i,l}-{\tilde{r}}_{i,l}^{c}}{\Gamma }$ are residues of $\left\lfloor \frac{X_{i}}{\Gamma }\right\rfloor$ modulo $M_{l}$ based on (7). So $q_{i}=\left\lfloor \frac{X_{i}}{\Gamma }\right\rfloor$. Subcase (c): Likewise, since $r_{i}^{c}+\Delta_{i,l}\in \left[\frac{3\Gamma }{4},\Gamma \right)$, we have ${\tilde{r}}_{i,l}^{c}={\langle r_{i}^{c}+\Delta_{i,l}\rangle }_{\Gamma }\in \left[\frac{3\Gamma }{4},\Gamma \right)$. Therefore, based on (A6), we have $q_{i}\equiv \frac{{\tilde{r}}_{i,l}-{\tilde{r}}_{i,l}^{c}}{\Gamma }+1$. From (7), one can obtain $\frac{{\tilde{r}}_{i,l}-{\tilde{r}}_{i,l}^{c}}{\Gamma }$ are residues of $\left\lfloor \frac{X_{i}}{\Gamma }\right\rfloor$ modulo $M_{l}$, which leads to $q_{i}=\left\lfloor \frac{X_{i}}{\Gamma }\right\rfloor +1$. Subcase (d): Since $r_{i}^{c}+\Delta_{i,l}\in \left(-\frac{\Gamma }{4},0\right)$, we have ${\tilde{r}}_{i,l}^{c}={\langle r_{i}^{c}+\Delta_{i,l}\rangle }_{\Gamma }\in \left(\frac{3\Gamma }{4},\Gamma \right)$. Therefore, based on (A6), we have $q_{i}\equiv \frac{{\tilde{r}}_{i,l}-{\tilde{r}}_{i,l}^{c}}{\Gamma }+1$. From (7), one can obtain $\frac{{\tilde{r}}_{i,l}-{\tilde{r}}_{i,l}^{c}}{\Gamma }$ are residues of $\lfloor \frac{X_{i}}{\Gamma }\rfloor -1$ modulo $M_{l}$. Thus, we have $q_{i}=\left\lfloor \frac{X_{i}}{\Gamma }\right\rfloor$. Subcase (e): Since $r_{i}^{c}+\Delta_{i,l}\in \left(\Gamma ,\frac{5\Gamma }{4}\right)$, we have ${\tilde{r}}_{i,l}^{c}={\langle r_{i}^{c}+\Delta_{i,l}\rangle }_{\Gamma }\in \left(0,\frac{\Gamma }{4}\right)$. Therefore, based on (A6), we have $q_{i}\equiv \frac{{\tilde{r}}_{i,l}-{\tilde{r}}_{i,l}^{c}}{\Gamma }$. From (7), one can obtain $\frac{{\tilde{r}}_{i,l}-{\tilde{r}}_{i,l}^{c}}{\Gamma }$ are residues of $\lfloor \frac{X_{i}}{\Gamma }\rfloor +1$ modulo $M_{l}$, which results in $q_{i}=\left\lfloor \frac{X_{i}}{\Gamma }\right\rfloor +1$. Since $|\Delta_{i,l}|<\Gamma /4$, only subcases (b) and (d) or subcases (c) and (e) can occur simultaneously. Clearly, subcases (b) and (d) result in the same $q_{i}$, i.e., $q_{i}=\left\lfloor \frac{X_{i}}{\Gamma }\right\rfloor$. Similarly, subcases c) and e) lead to the same $q_{i}$, where $q_{i}=\left\lfloor \frac{X_{i}}{\Gamma }\right\rfloor +1$. Based on the discussions above, we start to consider the residue pair $\left\{r_{1}^{c},r_{2}^{c}\right\}$. Since $r_{2}^{c}=\Gamma -r_{1}^{c}$, $\left\{r_{1}^{c},r_{2}^{c}\right\}$ must fall into one the four subcases:

(1) $r_{1}^{c}\in \left(0,\frac{\Gamma }{4}\right)$, $r_{2}^{c}\in \left(\frac{3\Gamma }{4},\Gamma \right)$
(2) $r_{1}^{c}\in \left(\frac{3\Gamma }{4},\Gamma \right)$, $r_{2}^{c}\in \left(0,\frac{\Gamma }{4}\right)$
(3) $r_{1}^{c}\in \left(\frac{\Gamma }{4},\frac{\Gamma }{2}\right)$, $r_{2}^{c}\in \left(\frac{\Gamma }{2},\frac{3\Gamma }{4}\right)$
(4) $r_{1}^{c}\in \left(\frac{\Gamma }{2},\frac{3\Gamma }{4}\right)$, $r_{2}^{c}\in \left(\frac{\Gamma }{4},\frac{\Gamma }{2}\right)$

Subcase (1): Since $r_{1}^{c}\in \left(0,\frac{\Gamma }{4}\right)$ and $|\Delta_{i,l}|<\frac{\Gamma }{4}$, $r_{1}^{c}+\Delta_{i,l}$ may fall into subcases (b) and (d), leading to $q_{1}=\left\lfloor \frac{X_{1}}{\Gamma }\right\rfloor$. In the same way, since $r_{2}^{c}\in \left(\frac{3\Gamma }{4},\Gamma \right)$, $r_{2}^{c}+\Delta_{i,l}$ can fall into subcases (c) and (e), resulting in $q_{2}=\left\lfloor \frac{X_{2}}{\Gamma }\right\rfloor +1$. Based on the fact that $x_{1}=-x_{2}$, we have $q_{2}=\left\lfloor \frac{X_{2}}{\Gamma }\right\rfloor +1=-\left\lfloor \frac{X_{1}}{\Gamma }\right\rfloor$, i.e., $q_{1}=-q_{2}=\left\lfloor \frac{X_{1}}{\Gamma }\right\rfloor$. Subcase (2): Subcase (2) is obviously symmetric with subcase (1) on the circle of length Γ. The proof is very similar to that of subcase (1). It is clear that $r_{1}^{c}+\Delta_{i,l}$ can fall into subcases (c) and (e), leading to $q_{1}=\left\lfloor \frac{X_{1}}{\Gamma }\right\rfloor +1$. $r_{2}^{c}+\Delta_{i,l}$ may fall into subcases (b) and (d), leading to $q_{2}=\left\lfloor \frac{X_{2}}{\Gamma }\right\rfloor$. Replacing $x_{2}$ with $-x_{1}$, we have $q_{2}=\left\lfloor \frac{X_{2}}{\Gamma }\right\rfloor =-\left\lfloor \frac{X_{1}}{\Gamma }\right\rfloor -1$. Thus, $q_{1}=-q_{2}=\left\lfloor \frac{X_{1}}{\Gamma }\right\rfloor +1$. Subcase (3): Since $r_{1}^{c}\in \left(\frac{\Gamma }{4},\frac{\Gamma }{2}\right)$ and $|\Delta_{i,l}|<\frac{\Gamma }{4}$, $r_{1}^{c}+\Delta_{i,l}$ may fall into subcases (b) and (d). Then, we have $q_{1}=\left\lfloor \frac{X_{1}}{\Gamma }\right\rfloor$. For $r_{2}^{c}\in \left(\frac{\Gamma }{2},\frac{3\Gamma }{4}\right)$, $r_{2}^{c}+\Delta_{i,l}$ can fall into subcases (c) and (e), resulting in $q_{2}=\left\lfloor \frac{X_{2}}{\Gamma }\right\rfloor +1$. Therefore, we have $q_{2}=\left\lfloor \frac{X_{2}}{\Gamma }\right\rfloor +1=-\left\lfloor \frac{X_{1}}{\Gamma }\right\rfloor$, i.e., $q_{1}=-q_{2}=\left\lfloor \frac{X_{1}}{\Gamma }\right\rfloor$. Subcase (4): Subcase (4) is clearly symmetric with subcase 3) on the circle of length Γ. Clearly, $r_{1}^{c}+\Delta_{i,l}$ can fall into subcases (c) and (e), leading to $q_{1}=\left\lfloor \frac{X_{1}}{\Gamma }\right\rfloor +1$. $r_{2}^{c}+\Delta_{i,l}$ may fall into subcases (b) and (d), which means $q_{2}=\left\lfloor \frac{X_{2}}{\Gamma }\right\rfloor$. Replacing $x_{2}$ with $-x_{1}$, we have $q_{2}=\left\lfloor \frac{X_{2}}{\Gamma }\right\rfloor =-\left\lfloor \frac{X_{1}}{\Gamma }\right\rfloor -1$. Thus, $q_{1}=-q_{2}=\left\lfloor \frac{X_{1}}{\Gamma }\right\rfloor +1$. In a nutshell, when Operation 2 is the appropriate operation, $q_{2}=-q_{1}$. If $r_{1}^{c}\in \left[0,\Gamma /2\right)$, $q_{1}=\left\lfloor \frac{X_{1}}{\Gamma }\right\rfloor$. Otherwise, $q_{1}=\left\lfloor \frac{X_{1}}{\Gamma }\right\rfloor +1$. Q.E.D. □

## Appendix C. Proof of Theorem 1

 **Proof.** At a high level, the proof is developed in two parts. First, we verify that $q_{1}$ has a unique solution when $X_{1}\in \left[0,\left\lfloor \sqrt{M}\right\rfloor \Gamma -\frac{\Gamma }{2}\right)$. Second, the reconstruction error is discussed under Operations 1 and 2, respectively. Now, we discuss the uniqueness of the solution to $q_{i}$. If Operation 1 is implemented, we have $q_{1}=\left\lfloor \frac{X_{1}}{\Gamma }\right\rfloor$ based on Lemma 2. Since $x_{1}<\left\lfloor \sqrt{M}\right\rfloor \Gamma -\frac{\Gamma }{2}$, we have

(A7) $$q_{1}=\left\lfloor \frac{X_{1}}{\Gamma }\right\rfloor <\left\lfloor \frac{\left\lfloor \sqrt{M}\right\rfloor \Gamma -\frac{\Gamma }{2}}{\Gamma }\right\rfloor =\left\lfloor \sqrt{M}\right\rfloor -1.$$

From Algorithm 1, $q_{1}$ is determined by (14) when Operation 1 is implemented. Clearly, $q_{1}^{2}+q_{1}$ in (14) satisfies $q_{1}^{2}+q_{1}<M$, which guarantees that $q_{1}^{2}+q_{1}$ can be uniquely determined via CRT. Once $q_{1}^{2}+q_{1}$ is known, $q_{1}$ can be uniquely determined by solving a quadratic equation. If Operation 2 is applied on ${\tilde{r}}_{i,l}^{c}$ and $r_{1}^{c}\in \left[0,\frac{\Gamma }{2}\right)$, we have $q_{1}=\left\lfloor \frac{X_{1}}{\Gamma }\right\rfloor$ based on Lemma 2, where $X_{1}\leq \left(\left\lfloor \sqrt{M}\right\rfloor -1\right)\Gamma +r_{1}^{c}$, i.e.,

(A8) $$q_{1}=\left\lfloor \frac{X_{1}}{\Gamma }\right\rfloor \leq \left\lfloor \frac{\left(\left\lfloor \sqrt{M}\right\rfloor -1\right)\Gamma +r_{1}^{c}}{\Gamma }\right\rfloor =\left\lfloor \sqrt{M}\right\rfloor -1.$$

If $r_{1}^{c}\in \left(\frac{\Gamma }{2},\Gamma \right)$ and Operation 2 is applied, we obtain $q_{1}=\left\lfloor \frac{X_{1}}{\Gamma }\right\rfloor +1$ based on Lemma 2. Correspondingly, $X_{1}\leq \left(\left\lfloor \sqrt{M}\right\rfloor -2\right)\Gamma +r_{1}^{c}$, which leads to

(A9) $$q_{1}=\left\lfloor \frac{X_{1}}{\Gamma }\right\rfloor +1\leq \left\lfloor \frac{\left(\left\lfloor \sqrt{M}\right\rfloor -2\right)\Gamma +r_{1}^{c}}{\Gamma }\right\rfloor +1=\left\lfloor \sqrt{M}\right\rfloor -1.$$

From Algorithm 1, $q_{1}$ is calculated by (13) when Operation 2 is implemented. Since $q_{1}\leq \left\lfloor \sqrt{M}\right\rfloor -1$, we obtain $q_{1}^{2}<M$ in (13), which means $q_{1}$ can be uniquely recovered. In the following, we continue to the second part of the proof, i.e., we discuss the robustness of reconstruction using the unique $q_{1}$, where $q_{1}\neq q_{2}$. Once $q_{1}$ is determined, we can obtain the corresponding ${\hat{r}}_{1,l}^{c}$ from (11) and further reconstruct ${\hat{X}}_{1}$ from (15). There are two scenarios for the reconstruction error: (i) Operation 1 is the appropriate operation and applied; (ii) Operation 2 is the appropriate operation and implemented. When Operation 1 is applied: In this case, ${\hat{r}}_{1,l}^{c}={\tilde{r}}_{1,l}^{c}$ based on (9). Then, we discuss ${\tilde{r}}_{1,l}^{c}={\langle r_{1}^{c}+\Delta_{1,l}\rangle }_{\Gamma }$, where $r_{1}^{c}+\Delta_{1,l}$ must fall into one of the three subcases:

(a) $r_{1}^{c}+\Delta_{1,l}\in \left(0,\Gamma \right)$
(b) $r_{1}^{c}+\Delta_{1,l}\in \left(-\frac{\Gamma }{4},0\right)$
(c) $r_{1}^{c}+\Delta_{1,l}\in \left(\Gamma ,\frac{5\Gamma }{4}\right)$

If $r_{1}^{c}+\Delta_{1,l}$ satisfies subcase (b), we have $\min_{il}d\left(0,{\tilde{r}}_{i,l}^{c}\right)<\frac{\Gamma }{4}<\min_{il}d\left(\frac{\Gamma }{2},{\tilde{r}}_{i,l}^{c}\right)$, a contradiction to the fact that Operation 1 is the appropriate operation. Likewise, when $r_{1}^{c}+\Delta_{1,l}$ falls into subcase (c), we have $\min_{il}d\left(0,{\tilde{r}}_{i,l}^{c}\right)<\frac{\Gamma }{4}<\min_{il}d\left(\frac{\Gamma }{2},{\tilde{r}}_{i,l}^{c}\right)$, which contradicts the fact that Operation 1 is the appropriate operation. Therefore, only subcase a) can happen, i.e., $r_{1}^{c}+\Delta_{1,l}\in \left(0,\Gamma \right)$. Thus,

$${\tilde{r}}_{1,l}^{c}={\langle r_{1}^{c}+\Delta_{1,l}\rangle }_{\Gamma }=r_{1}^{c}+\Delta_{1,l}.$$

Since Operation 1 is applied on ${\tilde{r}}_{1,l}^{c}$, we have $q_{1}=\left\lfloor \frac{X_{1}}{\Gamma }\right\rfloor$ based on Lemma 2. Then, reconstructing $X_{1}$ from (15), we have $|{\hat{X}}_{1}-X_{1}|$ is equal to

(A10) $$|\left\lfloor \frac{X_{1}}{\Gamma }\right\rfloor \Gamma +\frac{\sum_{l=1}^{L}{\hat{r}}_{1,l}^{c}}{L}-X_{1}\left|=\right|\left\lfloor \frac{X_{1}}{\Gamma }\right\rfloor \Gamma +\frac{\sum_{l=1}^{L}\left(r_{1}^{c}+\Delta_{1,l}\right)}{L}-X_{1}\left|=\right|\frac{\sum_{l=1}^{L}\Delta_{1,l}}{L}|<\frac{\Gamma }{4}.$$

When Operation 2 is applied: In this case, ${\hat{r}}_{1,l}^{c}={\tilde{r}}_{1,l}^{c}$ when ${\tilde{r}}_{1,l}^{c}\in \left[0,\Gamma /2\right)$; otherwise ${\hat{r}}_{1,l}^{c}={\tilde{r}}_{1,l}^{c}-\Gamma$ based on (9). Similarly, we discuss ${\tilde{r}}_{1,l}^{c}={\langle r_{1}^{c}+\Delta_{1,l}\rangle }_{\Gamma }$, where $r_{1}^{c}+\Delta_{1,l}$ must fall into one of the five subcases:

(a) $r_{1}^{c}+\Delta_{1,l}\in \left(\frac{\Gamma }{4},\frac{3\Gamma }{4}\right)$
(b) $r_{1}^{c}+\Delta_{1,l}\in \left[0,\frac{\Gamma }{4}\right]$
(c) $r_{1}^{c}+\Delta_{1,l}\in \left[\frac{3\Gamma }{4},\Gamma \right)$
(d) $r_{1}^{c}+\Delta_{1,l}\in \left(-\frac{\Gamma }{4},0\right)$
(e) $r_{1}^{c}+\Delta_{1,l}\in \left(\Gamma ,\frac{5\Gamma }{4}\right)$

If $r_{1}^{c}+\Delta_{1,l}$ satisfies subcase (a), we have $\min_{il}d\left(0,{\tilde{r}}_{i,l}^{c}\right)>\frac{\Gamma }{4}>\min_{il}d\left(\frac{\Gamma }{2},{\tilde{r}}_{i,l}^{c}\right)$, a contradiction to that Operation 2 is the appropriate operation, which means that subcase (a) cannot happen. As proved in Appendix B, when $r_{1}^{c}+\Delta_{1,l}$ falls into subcase (b) or (d), we have $q_{1}=\left\lfloor \frac{X_{1}}{\Gamma }\right\rfloor$. When $r_{1}^{c}+\Delta_{1,l}$ falls into subcase (c) or (e), we obtain $q_{1}=\left\lfloor \frac{X_{1}}{\Gamma }\right\rfloor +1$. Based on these conclusions, we discuss the reconstruction error: (i). only one subcase occurs; (ii). subcases (b) and (d) or subcases (c) and (e) happen simultaneously. Subcase (b): Since $r_{1}^{c}+\Delta_{1,l}\in \left[0,\frac{\Gamma }{4}\right]$ and Operation 2 is applied, we have ${\hat{r}}_{1,l}^{c}={\tilde{r}}_{1,l}^{c}={\langle r_{1}^{c}+\Delta_{1,l}\rangle }_{\Gamma }=r_{1}^{c}+\Delta_{1,l}$. Since $q_{1}=\left\lfloor \frac{X_{1}}{\Gamma }\right\rfloor$, $|{\hat{X}}_{1}-X_{1}|$ is equal to

(A11) $$|\left\lfloor \frac{X_{1}}{\Gamma }\right\rfloor \Gamma +\frac{\sum_{l=1}^{L}{\hat{r}}_{1,l}^{c}}{L}-X_{1}\left|=\right|\left\lfloor \frac{X_{1}}{\Gamma }\right\rfloor \Gamma +\frac{\sum_{l=1}^{L}\left(r_{1}^{c}+\Delta_{1,l}\right)}{L}-X_{1}\left|=\right|\frac{\sum_{l=1}^{L}\Delta_{1,l}}{L}|<\frac{\Gamma }{4}.$$

Subcase (c): Since $r_{1}^{c}+\Delta_{1,l}\in \left[\frac{3\Gamma }{4},\Gamma \right)$ and Operation 2 is applied, we have ${\hat{r}}_{1,l}^{c}={\tilde{r}}_{1,l}^{c}-\Gamma ={\langle r_{1}^{c}+\Delta_{1,l}\rangle }_{\Gamma }-\Gamma =r_{1}^{c}+\Delta_{1,l}-\Gamma$. Since $q_{1}=\left\lfloor \frac{X_{1}}{\Gamma }\right\rfloor +1$, $|{\hat{X}}_{1}-X_{1}|$ is equal to

(A12) $$|\left(\left\lfloor \frac{X_{1}}{\Gamma }\right\rfloor \;+\;1\right)\Gamma \;+\;\frac{\sum_{l=1}^{L}{\hat{r}}_{1,l}^{c}}{L}\;-\;X_{1}\left|=\right|\left\lfloor \frac{X_{1}}{\Gamma }\right\rfloor \Gamma \;+\;\Gamma \;+\;\frac{\sum_{l=1}^{L}\left(r_{1}^{c}\;+\;\Delta_{1,l}\;-\;\Gamma \right)}{L}\;-\;X_{1}\left|=\right|\frac{\sum_{l=1}^{L}\Delta_{1,l}}{L}|<\frac{\Gamma }{4}.$$

Subcase (d): Since $r_{1}^{c}+\Delta_{1,l}\in \left(-\frac{\Gamma }{4},0\right)$ and Operation 2 is applied, we have ${\hat{r}}_{1,l}^{c}={\tilde{r}}_{1,l}^{c}-\Gamma ={\langle r_{1}^{c}+\Delta_{1,l}\rangle }_{\Gamma }-\Gamma =r_{1}^{c}+\Delta_{1,l}$. Since $q_{1}=\left\lfloor \frac{X_{1}}{\Gamma }\right\rfloor$, $|{\hat{X}}_{1}-X_{1}|$ is equal to

(A13) $$|\left\lfloor \frac{X_{1}}{\Gamma }\right\rfloor \Gamma +\frac{\sum_{l=1}^{L}{\hat{r}}_{1,l}^{c}}{L}-X_{1}\left|=\right|\left\lfloor \frac{X_{1}}{\Gamma }\right\rfloor \Gamma +\frac{\sum_{l=1}^{L}\left(r_{1}^{c}+\Delta_{1,l}\right)}{L}-X_{1}\left|=\right|\frac{\sum_{l=1}^{L}\Delta_{1,l}}{L}|<\frac{\Gamma }{4}.$$

Subcase (e): Since $r_{1}^{c}+\Delta_{1,l}\in \left(\Gamma ,\frac{5\Gamma }{4}\right)$ and Operation 2 is applied, we have ${\hat{r}}_{1,l}^{c}={\tilde{r}}_{1,l}^{c}={\langle r_{1}^{c}+\Delta_{1,l}\rangle }_{\Gamma }=r_{1}^{c}+\Delta_{1,l}-\Gamma$. Since $q_{1}=\left\lfloor \frac{X_{1}}{\Gamma }\right\rfloor +1$, $|{\hat{X}}_{1}-X_{1}|$ is equal to

(A14) $$|\left(\left\lfloor \frac{X_{1}}{\Gamma }\right\rfloor +1\right)\Gamma \;+\;\frac{\sum_{l=1}^{L}{\hat{r}}_{1,l}^{c}}{L}\;-\;X_{1}\left|=\right|\left\lfloor \frac{X_{1}}{\Gamma }\right\rfloor \Gamma \;+\;\Gamma \;+\;\frac{\sum_{l=1}^{L}\left(r_{1}^{c}\;+\;\Delta_{1,l}\;-\;\Gamma \right)}{L}\;-\;X_{1}\left|=\right|\frac{\sum_{l=1}^{L}\Delta_{1,l}}{L}|<\frac{\Gamma }{4}.$$

Subcase (b) and (d) happen simultaneously: Based on the discussions above, we have $q_{1}=\left\lfloor \frac{X_{1}}{\Gamma }\right\rfloor$ and ${\hat{r}}_{1,l}^{c}=r_{1}^{c}+\Delta_{1,l}$. Therefore, $|{\hat{X}}_{1}-X_{1}|$ is equal to

(A15) $$|\left\lfloor \frac{X_{1}}{\Gamma }\right\rfloor \Gamma +\frac{\sum_{l=1}^{L}{\hat{r}}_{1,l}^{c}}{L}-X_{1}\left|=\right|\left\lfloor \frac{X_{1}}{\Gamma }\right\rfloor \Gamma +\frac{\sum_{l=1}^{L}\left(r_{1}^{c}+\Delta_{1,l}\right)}{L}-X_{1}\left|=\right|\frac{\sum_{l=1}^{L}\Delta_{1,l}}{L}|<\frac{\Gamma }{4}.$$

Subcase (c) and (e) happen simultaneously: Likewise, based on the discussions above, we have $q_{1}=\left\lfloor \frac{X_{1}}{\Gamma }\right\rfloor +1$ and ${\hat{r}}_{1,l}^{c}=r_{1}^{c}+\Delta_{1,l}-\Gamma$. As a result, $|{\hat{X}}_{1}-X_{1}|$ is equal to

(A16) $$|\left(\left\lfloor \frac{X_{1}}{\Gamma }\right\rfloor \;+\;1\right)\Gamma \;+\;\frac{\sum_{l=1}^{L}{\hat{r}}_{1,l}^{c}}{L}\;-\;X_{1}\left|=\right|\left\lfloor \frac{X_{1}}{\Gamma }\right\rfloor \Gamma \;+\;\Gamma \;+\;\frac{\sum_{l=1}^{L}\left(r_{1}^{c}\;+\;\Delta_{1,l}\;-\;\Gamma \right)}{L}\;-\;X_{1}\left|=\right|\frac{\sum_{l=1}^{L}\Delta_{1,l}}{L}|<\frac{\Gamma }{4}.$$

Thus, the reconstruction error is bounded by $\frac{\Gamma }{4}$. Q.E.D. □
